# Multiple house occupancy is associated with mortality in hospitalized patients with COVID-19

**DOI:** 10.1093/eurpub/ckab085

**Published:** 2021-05-17

**Authors:** Eilidh Bruce, Ben Carter, Terence J Quinn, Alessia Verduri, Oliver Pearson, Arturo Vilches-Moraga, Angeline Price, Aine McGovern, Louis Evans, Kathryn McCarthy, Jonathan Hewitt, Susan Moug, Phyo K Myint, COPE Study Team

**Affiliations:** 1 Institute of Applied Health Sciences, University of Aberdeen, Aberdeen, UK; 2 Department of Biostatistics & Health Informatics, King’s College London, London, UK; 3 University of Glasgow, Glasgow, UK; 4 University of Modena and Reggio Emilia - Hospital Policlinico Modena, Modena, Italy; 5 Salford Royal NHS Trust, Salford, UK; 6 Manchester University, Manchester, UK; 7 Glasgow Royal Infirmary, Glasgow, UK; 8 Ysbyty Ystrad Fawr, Aneurin Bevan University Health Board, Wales, UK; 9 North Bristol NHS Trust, Bristol, UK; 10 Cardiff University, Cardiff, Wales, UK; 11 Department of Medicine for the Elderly, NHS Grampian, Aberdeen, UK

## Abstract

**Background:**

In response to the COVID-19 pandemic, many countries mandated staying at home to reduce transmission. This study examined the association between living arrangements (house occupancy numbers) and outcomes in COVID-19.

**Methods:**

Study population was drawn from the COPE study, a multicentre cohort study. House occupancy was defined as: living alone; living with one other person; living with multiple other people; or living in a nursing/residential home. Outcomes were time from admission to mortality and discharge (Cox regression), and Day 28 mortality (logistic regression) analyses were adjusted for key comorbidities and covariates including admission: age, sex, smoking, heart failure, admission C-reactive protein (CRP), chronic obstructive pulmonary disease, estimated glomerular filtration rate, frailty and others.

**Results:**

A total of 1584 patients were included from 13 hospitals across UK and Italy: 676 (42.7%) were female, 907 (57.3%) were male, median age was 74 years (range: 19–101). At 28 days, 502 (31.7%) had died. Median admission CRP was 67, 82, 79.5 and 83 mg/l for those living alone, with someone else, in a house of multiple occupancy and in a nursing/residential home, respectively. Compared to living alone, living with anyone was associated with increased mortality: within a couple [adjusted hazard ratios (aHR) = 1.39, 95% confidence intervals (CI) 1.09–1.77, *P* = 0.007]; living in a house of multiple occupancy (aHR = 1.67, 95% CI 1.17–2.38, *P* = 0.005); and living in a residential home (aHR = 1.36, 95% CI 1.03–1.80, *P* = 0.031).

**Conclusion:**

For patients hospitalized with COVID-19, those living with one or more people had an increased association with mortality, they also exhibited higher CRP indicating increased disease severity suggesting they delayed seeking care.

## Introduction

The COVID-19 pandemic has provided one of the greatest challenges known to public health. As of 15 December 2020, there have been over 70 million cases and 1.6 million deaths attributed to the virus worldwide.^1^ Transmission of the SARS-CoV-2 virus is dependent on human interaction and behaviours, and this has been the single most important public health target across the globe.^2^ In addition to handwashing^3^ and the use of face-coverings,^4^ the public have been encouraged to physically distance and ‘Stay at Home’ throughout. Furthermore, current UK law requires people to self-isolate if they or someone they live with are symptomatic or have a positive test.^5^ More specifically, those infected with COVID-19 who live with another person or in a house of multiple occupancy are advised to self-isolate within their homes separate from other household members. This has been effective from a public health perspective in suppressing transmission of the virus.

However, it is not known whether outcomes in COVID-19 disease may be affected by social determinants such as living arrangements. Whilst isolation measures may reduce transmission, evidence is lacking with regard to how the environment in which people are isolating impacts on the severity of disease and outcomes for individuals who have contracted COVID-19. There has been no study to date that examines patient outcomes in relation to house occupancy. The primary aim of this study is to examine the association between living arrangements and in-hospital mortality in patients with COVID-19 infection, the secondary aim was to assess occupancy on time-to-discharge and disease severity estimated by serum C-reactive protein (CRP) levels.

## Methods

### Study design

Data were collated as part of a European multicentre observational study: COPE (*C*OVID-19 in *O*lder *Pe*ople study).^6^ The protocol has been published elsewhere.^7^ Ethical approval was obtained in the UK by the Health Research Authority (20/HRA/1898) and the Ethics Committee of Policlinico Hospital Modena, Italy (369/2020/OSS/AOUMO), respectively. This study has been reported following the STROBE statement.^8^ A central MACRO database, hosted by King’s Clinical Trials Unit, was used to collate the data centrally.

### Setting

The study sites included an established network of 12 UK sites and 1 Italian site. The UK centres included Aberdeen Royal Infirmary, Glasgow Royal Infirmary, Inverclyde Royal Hospital, Maidstone Hospital, Nevill Hall Hospital in Abergavenny, Royal Alexandra Hospital in Paisley, Royal Gwent Hospital in Newport, Southmead Hospital in Bristol, Salford Royal Hospital, University Hospital of Wales in Cardiff, Ysbyty Gwynedd in Bangor and Ysbyty Ystrad Fawr in Caerphilly. The Italian centre was the University Hospital of Modena Policlinico.

### Participants

Each site research team screened hospital admission lists daily between 27 February 2020 and 10 June 2020. The ethical approval was such that formal written consent from participants was not required as all data were routinely collected in hospital records.

#### Inclusion/exclusion criteria

The study included consecutive hospitalized adult patients aged 18 years or older with a confirmed diagnosis of COVID-19 admitted between 27 February 2020 and 10 June 2020; diagnostic criteria included laboratory-confirmed SARS-CoV-2 positive swab or a clinical diagnosis of COVID-19; patients needed to be followed up at least for 28 days or until death.

### Outcome

The primary outcome was length of time from admission to mortality. Secondary outcomes were the time-to-discharge, and Day 28 mortality. Patients who were discharged prior to Day 28 were censored at this point in the time to mortality analysis and assumed to be alive in the Day 28 mortality analysis, and those died were censored in the time to discharge analysis. Those alive and still in hospital with less than 28 days follow-up were excluded from the Day 28 mortality analysis. For patients diagnosed with COVID-19 whilst as an inpatient the date of diagnosis was used rather than date of admission to hospital. Other prespecified outcomes included in the COPE study were the effect of drug classes, nosocomial infection and frailty, however, these are not analysed here and have been reported in previous publications.^6^^,^^9–11^

### Primary exposure

Home occupancy was categorized as follows: living alone; living with one other person; living in a house of multiple occupancy (not in a residential or nursing home); and living in a residential or nursing care home.

### Covariates

Demographic and clinical characteristics recorded at admission were age, sex, smoking status (never, previous or current), CRP as a marker of disease severity (REF Stringer et al., under revision with Int J Epidemiol), estimated glomerular filtration rate (eGFR), previous history of coronary artery disease (CAD), diabetes mellitus, chronic obstructive pulmonary disease (COPD), hypertension (no, yes not on treatment and yes on treatment), albumin, heart failure and frailty (using the Clinical Frailty Scale; CFS).

### Data analysis

Admission demographic and clinical characteristics were presented by Day 28 mortality, to describe the included participant characteristics.

Time to mortality (primary outcome) and length of stay (secondary outcome) were analysed with mixed-effects multivariable Cox’s proportional baseline hazards regression models. The analyses were fitted with a random effect to account for hospital variation (26), and adjusted for the base model of patient age group; sex; smoking status; CRP; diabetes; hypertension; eGFR; COPD; CAD; albumin; heart failure; and frailty (CFS; 1–3 versus 4–5 and 6–9). The adjusted hazard ratios (aHR) were estimated with associated 95% confidence intervals (95% CI). The baseline proportionality assumption was tested visually with log–log residuals. Each time to event analysis was reported with a Kaplan–Meier survival plot.

The secondary outcome of Day 28 mortality was analysed using a mixed-effects multivariable logistic model, fitting each hospital as a random intercept effect, and adjusted with covariates consistent with the primary outcome. The adjusted odds ratio (OR) were presented with associated 95% CI. Missing data were explored for patterns of missingness. Analysis was carried out using Stata/MP version 16.0.

Deprivation, using the Index of Multiple Deprivation (IMD 1–3 versus 4–7 and 8–10) was additionally fitted to the primary analysis of the mixed-effects multivariable Cox proportional hazards (Cox PH analysis as a sensitivity analysis.

## Results

The study included 1671 patients from 13 hospitals; however, 87 (5.2%) did not have 28 days follow-up (or mortality) and were excluded. Of the 1584 included patients, 676 (42.7%) were female, 907 (57.3%) were male, median age was 74 years [interquartile range (IQR): 61.5–83; range: 19–101] and 1417 (89.5%) were white (table 1). With regard to comorbidities, 38.5% were taking medication for hypertension, and 60.0% had low albumin. In-hospital mortality in patients with at least 28-day follow-up (or who died) was 31.7% (502/1584). There were 433 patients who lived alone and 32.1% died, compared to 29.4% who died and lived with one other. Of 189 patients who lived in house of multiple occupancy outside of a nursing or residential home 25.4% died, compared to 47.0% who lived in a nursing or residential home. There were 141 cases (8.9%) where housing occupancy was not reported.

**Table 1:** Demographics and comorbidities for the included patients

	Mortality at Day 28		
	Alive (*N* = 1082)	Dead (*N* = 502)	Total (*N* = 1584)
Sex			
Female	468 (69.2)	208 (30.8)	676 (42.7)
Male	613 (67.6)	294 (32.4)	907 (57.3)
Missing	1	0	1
Age			
≤64	420 (87.3)	61 (12.7)	481 (30.4)
65–74	221 (70.8)	91 (29.2)	312 (19.7)
75–84	257 (57.1)	193 (42.9)	450 (28.4)
85+	184 (54.0)	157 (46.0)	341 (21.5)
Ethnicity			
White	957 (67.5)	460 (32.5)	1417 (89.5)
Asian/Asian British	32 (68.1)	15 (31.9)	47 (3.0)
Black/Black British	17 (77.3)	5 (22.7)	22 (1.4)
Chinese	3 (75.0)	1 (25.0)	4 (0.3)
Mixed	1 (100.0)	0 (0.0)	1 (0.1)
Other	11 (91.7)	1 (8.3)	12 (0.8)
Missing	61	20	81
Living arrangement			
Lives alone	294 (67.9)	139 (32.1)	433 (27.3)
Lives with 1 other	425 (70.6)	177 (29.4)	602 (38)
Lives with multiple others (not in a residential or nursing home)	141 (74.6)	48 (25.4)	189 (11.9)
Lives with multiple others (in a residential or nursing home)	116 (53.0)	103 (47.0)	219 (13.8)
Missing	106	35	141
Smoking			
Never smoked	587 (71.2)	237 (28.8)	824 (52.0)
Ex-smoker	381 (63.0)	224 (37.0)	605 (38.2)
Current smoker	85 (75.9)	27 (24.1)	112 (7.1)
Missing	29	14	43
Diabetes			
No	812 (69.7)	353 (30.3)	1165 (73.6)
Yes	268 (64.4)	148 (35.6)	416 (26.3)
Missing	2	1	3
Coronary artery disease			
No	896 (71.6)	356 (28.4)	1252 (79.0)
Yes	185 (56.1)	145 (43.9)	330 (20.8)
Missing	1	1	2
Hypertension			
No	560 (70.1)	239 (29.9)	799 (50.4)
Yes	112 (64.7)	61 (35.3)	173 (10.9)
Yes (on treatment)	409 (67.2)	200 (32.8)	609 (38.5)
Missing	1	2	3
CRP			
<40	380 (82.3)	82 (17.8)	462 (29.2)
≥40	689 (62.9)	406 (37.1)	1095 (69.1)
Missing	13	14	27
eGFR			
≥60	745 (75.7)	239 (24.3)	984 (62.1)
45–59	120 (61.5)	75 (38.5)	195 (12.3)
30–44	105 (54.4)	88 (45.6)	193 (12.2)
<30	91 (53.9)	78 (46.2)	169 (10.7)
Missing	21	22	43
Albumin			
≥35	425 (76.2)	133 (23.8)	558 (35.2)
<35	606 (63.7)	345 (36.3)	951 (60.0)
Missing	51	24	75
COPD			
No	914 (70.1)	390 (29.9)	1304 (82.3)
Yes	116 (57.7)	85 (42.3)	201 (12.7)
Missing	52	27	79
Heart failure			
No	940 (70.4)	396 (29.6)	1336 (84.3)
Yes	90 (54.2)	76 (45.8)	166 (10.5)
Missing	52	30	82
Clinical Frailty Scale			
1–3	468 (83.4)	93 (16.6)	561 (35.4)
4–5	257 (68.2)	120 (31.8)	377 (23.8)
6–9	351 (55.3)	284 (44.7)	635 (40.0)
Missing	6	5	11

**Figure 1:**
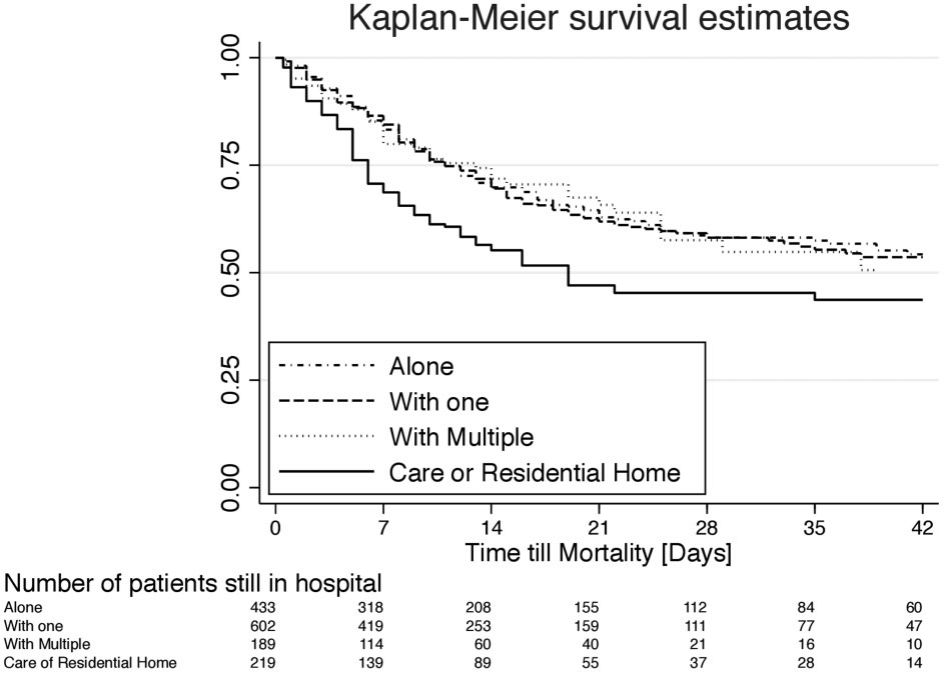
Kaplan–Meier depiction of overall survival in association with living arrangements.

The Kaplan–Meier overall survival plot demonstrates the association between residential or nursing care and mortality (figure 1). However, little differences are suggested from the other three house occupancy groups. The demographic and clinical characteristics of the house occupancy distribution show that patients who live alone were more likely to be older, than those living in a couple of in a house of multiple occupancy (Supplementary table S1). The admission CRP for those living alone was median = 67 mg/l (28–130 IQR), compared to those living as a couple, median = 82 mg/l (34–155 IQR), or living in a house of multiple occupancy median = 79.5 mg/l (45–150 IQR) or living in a nursing home median CRP = 83 mg/l (35–138 IQR).

### Data analysis

Within the crude mixed-effects Cox regression analysis, there was an association between living alone versus living in a care home, and mortality HR = 1.60 (95% CI 1.24–2.05; *P* < 0.001), the full set of crude analyses can be found in table 2.

**Table 2:** Crude and multivariable cox proportional hazards regression presenting crude and adjusted^a^ HR analysis of the time to mortality

	Crude HR (95% CI)	*P*-value	Adjusted HR^a^ (95% CI)	*P*-value
Sex				
Female	Reference		Reference	
Male	1.05 (0.88–1.25)	0.579	1.21 (0.99–1.48)	0.060
Age				
<65	Reference		Reference	
65–74	2.33 (1.70–3.20)	<0.001	1.85 (1.47–2.68)	0.001
75–84	3.68 (2.78–4.88)	<0.001	2.93 (2.04–4.21)	<0.001
85+	3.85 (2.88–5.14)	<0.001	3.08 (2.10–4.54)	<0.001
Smoking				
Never smoked	Reference		Reference	
Ex-smoker	1.26 (1.05–1.50)	0.012	1.00 (0.82–1.24)	0.867
Current smoker	0.76 (0.51–1.12)	0.169	0.93 (0.61–1.41)	0.725
Diabetes				
No	Reference		Reference	
Yes	1.11 (0.92–1.34)	0.288	1.05 (0.85–1.30)	0.630
Coronary artery disease				
No	Reference		Reference	
Yes	1.57 (1.29–1.90)	<0.001	1.13 (0.90–1.41)	0.307
Hypertension				
No	Reference		Reference	
Yes	1.12 (0.85–1.49)	0.412	0.90 (0.66–1.24)	0.522
Yes (on treatment)	1.15 (0.95–1.38)	0.150	0.91 (0.74–1.13)	0.414
CRP				
<40	Reference		Reference	
≥40	1.92 (1.55–2.38)	<0.001	2.04 (1.61–2.60)	<0.001
eGFR				
≥60	Reference		Reference	
45–59	1.77 (1.37–2.29)	<0.001	1.48 (1.12–1.95)	0.006
30–44	2.06 (1.62–2.62)	<0.001	1.48 (1.13–1.95)	0.004
<30	1.83 (1.42–2.37)	<0.001	1.50 (1.13–1.97)	0.004
Albumin				
≥35	Reference		Reference	
<35	1.45 (1.17–1.78)	0.001	1.34 (1.06–1.70)	0.014
COPD				
No	Reference		Reference	
Yes	1.58 (1.26–1.99)	<0.001	1.31 (1.01–1.70)	0.039
Heart failure				
No	Reference		Reference	
Yes	1.66 (1.30–2.12)	<0.001	1.09 (0.83–1.44)	0.528
Clinical Frailty Scale				
1–3	Reference		Reference	
4–5	1.70 (1.30–2.21)	<0.001	1.18 (0.86–1.64)	0.307
6–9	2.45 (1.94–3.11)	<0.001	1.49 (1.09–2.05)	0.013
Living arrangement				
Lives alone	Reference		Reference	
Lives with one other	1.05 (0.84–1.31)	0.691	1.39 (1.09–1.77)	0.007
Lives with multiple others (not in a residential or nursing home)	0.97 (0.69–1.34)	0.836	1.67 (1.17–2.38)	0.005
Lives with multiple others (in a residential or nursing home)	1.60 (1.24–2.05)	<0.001	1.36 (1.03–1.80)	0.031

*Note*: The number of observations excluded due to missing data were 227.

a Multivariable analysis was adjusted by sex, age, smoking, diabetes, coronary artery disease, hypertension, CRP, eGFR, albumin, COPD, heart failure and Clinical Frailty Scale.

Within the adjusted multivariable Cox regression, house occupancy was associated with mortality. Compared to living alone, co-habiting was associated with increased mortality such as within in a couple (aHR = 1.39, 95% CI 1.09–1.77, *P* = 0.007); living in a house of multiple occupancy (aHR = 1.67, 95% CI 1.17–2.38, *P* = 0.005); and living in a nursing or residential home (aHR = 1.36, 95% CI 1.03–1.80, *P* = 0.031). Other important covariates in associated with mortality in the adjusted analysis were age, CRP, eGFR, albumin and COPD, full details can be found in table 2.

In the secondary outcome 28-day mortality, occupancy was associated with mortality for patients in a nursing or residential home OR = 1.88 (95% CI 1.34–2.64, *P* < 0.001) (table 3). Other covariates associated with morality in this outcome were age, smoking, CAD, CRP, eGFR, albumin, COPD and CHF (table 3). In the secondary outcome of time to discharge there was a crude association between having someone at home and increased discharge (compared to living alone), living as a couple (HR = 1.31, 95% CI 1.11–1.55, *P* = 0.002) and living with multiple others (HR = 1.56, 95% CI 1.25–1.95, *P* < 0.001) (Supplementary table S2). In the adjusted multivariable analysis, there was an association between living alone versus in a residential/nursing home, and time to discharge (aHR = 1.41, 95% CI 1.08–1.84, *P* = 0.012).

**Table 3:** Crude and multivariable logistic regression presenting crude and adjusted multivariable^a^ OR for mortality at Day 28

	Crude OR (95% CI)	*P*-value	Adjusted OR^a^ (95% CI)	*P*-value
Sex				
Female	Reference		Reference	
Male	1.12 (0.90–1.40)	0.310	1.44 (1.09–1.90)	0.010
Age				
≤64	Reference		Reference	
65–74	3.28 (2.26–4.76)	<0.001	2.17 (1.38–3.39)	0.001
75–84	6.26 (4.43–8.83)	<0.001	4.20 (2.69–6.55)	<0.001
85+	7.53 (5.22–10.87)	<0.001	4.96 (3.03–8.12)	<0.001
Smoking				
Never smoked	Reference		Reference	
Ex-smoker	1.43 (1.14–1.79)	0.002	1.06 (0.79–1.42)	0.690
Current smoker	0.72 (0.45–1.15)	0.174	0.99 (0.57–1.75)	0.985
Diabetes				
No	Reference		Reference	
Yes	1.20 (0.94–1.53)	0.144	1.06 (0.78–1.43)	0.725
Coronary artery disease				
No	Reference		Reference	
Yes	1.97 (1.52–2.55)	<0.001	1.17 (0.84–1.61)	0.349
Hypertension				
No	Reference		Reference	
Yes	1.21 (0.84–1.73)	0.306	0.80 (0.51–1.25)	0.322
Yes (on treatment)	1.24 (0.98–1.57)	0.075	0.94 (0.69–1.26)	0.661
CRP				
<40	Reference		Reference	
≥40	2.50 (1.92–3.25)	<0.001	2.60 (1.90–3.56)	<0.001
eGFR				
≥60	Reference		Reference	
45–59	2.05 (1.47–2.87)	<0.001	1.38 (0.94–2.03)	0.098
30–44	2.62 (1.89–3.64)	<0.001	1.41 (0.95–2.10)	0.087
<30	2.46 (1.74–3.47)	<0.001	1.81 (1.19–2.73)	0.005
Albumin				
≥35	Reference		Reference	
<35	2.35 (1.79–3.08)	<0.001	1.88 (1.36–2.60)	<0.001
COPD				
No	Reference		Reference	
Yes	1.72 (1.26–2.35)	0.001	1.32 (0.90–1.95)	0.155
Heart failure				
No	Reference		Reference	
Yes	2.07 (1.47–2.90)	<0.001	1.08 (0.72–1.64)	0.698
Frailty				
1–3	Reference		Reference	
4–5	2.37 (1.72–3.26)	<0.001	1.29 (0.86–1.94)	0.226
6–9	4.46 (3.32–5.99)	<0.001	2.26 (1.51–3.41)	<0.001
Living arrangement				
Lives alone	Reference		Reference	
Lives with 1 other	0.91 (0.69–1.20)	0.505	1.34 (0.97–1.87)	0.077
Lives with multiple others (not in a residential or nursing home)	0.70 (0.47–1.05)	0.085	1.46 (0.90–2.35)	0.121
Lives with multiple others (in a residential or nursing home)	1.88 (1.34–2.64)	<0.001	1.37 (0.91–2.06)	0.134

*Note*: The number of observations excluded due to missing data were 227.

a Multivariable analysis was adjusted by age, sex, smoking, diabetes, coronary artery disease, hypertension, CRP, eGFR, albumin, COPD, heart failure and frailty.

After additionally adjusting for deprivation using the IMD (1–3, versus 4–7, and 8–10), there was no change in the effect of occupancy, and both the magnitude and significance of each finding were maintained.

## Discussion

Our study reports the novel association between home living arrangements and outcomes in hospitalized patients with COVID-19. In this large prospective study, we have provided further evidence on the association of residential/care home living and increased mortality from the disease. However, we also present the novel finding that multiple house occupancy (i.e. living with either one or multiple other people) is associated with increased mortality in COVID-19 infection in community settings.

The association between nursing home residents and mortality in COVID-19 is well described. Although less than 0.5% of the total population of the USA live in nursing homes, nursing home residents have accounted for around 25% of the documented deaths in COVID-19.^12^ A number of American states published their individual data during the early months of the pandemic. This has shown that deaths in long-term care facilities have accounted for over 50% of all COVID-19 deaths in the states of Delaware, Massachusetts, Oregon, Pennsylvania, Colorado and Utah.^13^

Our findings may be explained by the effect of public health messages delaying individual patients presenting to hospital. If incapacitated by the virus, those living alone may be less able to cope with their symptoms and the difficulties caused by self-isolation, therefore more likely to exhibit health seeking behaviour and thus present to hospital earlier with less severe disease. This could explain the lower admission CRP levels and subsequent lower mortality risk seen in this patient group. Conversely, those living with others may be encouraged to remain at home for longer despite a worsening clinical state and are possible to do so by support of other household members.

There are a number of plausible theories that may explain the observed associations we found. A large prospective study has shown an independent relationship between high viral load and mortality, adjusted for age, sex and common comorbidities.^14^ If this evidence is considered in the context of living arrangements, those who live with others are at risk of contracting the virus from another member of their household. In the lockdown climate of the early pandemic (when COPE was conducted), the public health message to stay at home rendered many households isolating together, exposing other members of the household to the virus if one were to contract it. It is therefore possible that those living in houses of multiple occupancy have increased viral load exposure and resultant increased mortality outcomes from COVID-19. Multiple house occupancy was also seen to be associated with raised CRP at admission, which can be interpreted as a proxy for increased disease severity.^15^ This is clinically relevant to public health policymakers. The message should be very clear that strict self-isolation for COVID-19 positive people within houses of multiple occupancy is vital in order to reduce virus exposure to other members of the household. It may be advisable to consider living alone as a vulnerable person to reduce risk of developing severe disease.

Indeed, these theories are supported by knowledge of health seeking behaviours and how they have been shaped by the COVID-19 pandemic. Fear and threat are central to the emotional responses felt by many during a pandemic.^16^ Negative framing of the pandemic from the media’s perspective often fuels this, e.g. by reporting the number of cases and deaths rather than the data on those who recover. Those who are not mathematically skilled with knowledge regarding probabilities and risk are particularly susceptible to fear as a result of negative framing.^17^ Fear and strict adherence to public health instruction to stay at home may have encouraged people to keep their unwell family members at home until they deteriorate to extremis. This could account for higher admission CRP and mortality risk in those from houses of multiple occupancy, observed in this study.

This was an observational study and is therefore is subject to the intrinsic limitations associated with all studies of this nature. The prospective nature of the data collection reduces the possibility of reverse causality, and the collection of unselected and consecutive data for all patients admitted with COVID-19 acts to reduce selection bias. The study was limited to those hospitalized, it also does not capture data from patients who remained in the community or patients who were discharged from (or died) the Emergency Department. The impact of confounding variables has also been addressed by statistical adjustment for variables including age, comorbidities and admission CRP. Patients with nosocomial COVID-19 infection (infection that is acquired by a patient who was admitted for another reason) were a small percentage of the study population and are unlikely to impact on the results as they have been found to have a lower mortality rate than patients with community acquired infection.^11^ We did not collect the duration of symptoms prior to admission and also did not collect the isolation arrangements between household members since onset of the symptoms, but this is of less relevance as it is well known that infectivity begins prior to symptom onset. We did not contact trace to household members and thus unable to uncertain to an extent that findings are contributed by infection from other household member who did not require hospital admission but contributed to potentially higher viral load depicted by higher CRP levels amongst those from homes with multiple occupancy. Information regarding dependency was not collected, and therefore it is also possible that these findings are confounded by the possibility that those people in houses of multiple occupancy are dependent and therefore at risk of worse outcomes. The analyses did not adjust for ethnicity because the majority of the sample was white (89.5%), therefore an analysis of ethnicity would be underpowered. We did not collect the detailed information on the treatment received by patients, but at the time of the study, the understanding of treatment of COVID-19 was sparse, and all centres are likely to have had similar approaches in management.

We found these patients from multiple occupancy households to have increased levels of CRP at time of presentation, a proxy marker for disease severity. This is novel information into the impact of living arrangements and outcomes in COVID-19 disease, during a pandemic where there has been much uncertainty. Public health measures, although effective at preventing overall disease transmission, should further highlight the importance of self-isolation within a household to reduce the possible effect of increased viral load. Messages should also encourage patients who are clinically deteriorating at home to present to hospital for appropriate treatment. We believe that the implications of these findings are relevant not only to the second wave of the current COVID-19 pandemic, but also to future public health crises of highly contagious diseases.

## Supplementary data

Supplementary data are available at *EURPUB* online.

*Conflicts of interest*: None declared.

**Key points**

- Multiple house occupancy (i.e. living with either one or multiple other people) is associated with increased mortality in COVID-19 infection in community settings.
- Patients from multiple occupancy households to have increased levels of CRP at time of presentation, a proxy marker for disease severity.
- Mortality risk in COVID-19 is multi-factorial and should be considered in the context of environmental circumstances as well as clinical and demographic variables.

## Supplementary Material

ckab085_Supplementary_Data

## Notes

**Study registration number:** IRAS project ID 281951.

**Approval numbers:** UK Health Research Authority (Reference: 20/HRA/1898).

Italy Ethics Committee of Policlinico Hospital Modena (Reference 369/2020/OSS/AOUMO).

**Protocol number:** SPON1815-20.
