# Supplementary material for: Multiple house occupancy is associated with mortality in hospitalized patients with COVID-19
Source: Eur J Public Health. 2021 May 17;32(1):133–9. doi: 10.1093/eurpub/ckab085 (PMC8247274; doi:10.1093/eurpub/ckab085)
Supplement: ckab085_Supplementary_Data [file ckab085_supplementary_data.zip › ejph-2021-01-om-0093-File003.docx]

Multiple House Occupancy is Associated with Mortality in Hospitalised Patients with Covid-19

Eilidh Bruce MBChB^1^, Ben Carter Ph.D.^2^, Terence J Quinn M.D.^3^, Alessia Verduri M.D.^4^, Oliver Pearson^2^, Arturo Vilches-Moraga F.R.C.P.^5^, Angeline Price M.Sc.^6^, Aine McGovern M.R.C.P.^7^, Louis Evans^8^, Kathryn McCarthy M.D.^9^, Jonathan Hewitt Ph.D.^10^, Susan Moug Ph.D.^11^, Phyo K Myint^#^ M.D.^1,12^ On behalf of COPE Study Team.

1. Institute of Applied Health Science, University of Aberdeen, Aberdeen Scotland
2. Department of Biostatistics &Health Informatics, King’s College London, England
3. University of Glasgow, Glasgow, Scotland
4. University of Modena and Reggio Emilia - Hospital Policlinico Modena, Italy
5. Salford Royal NHS Trust and, Salford
6. Manchester University, Manchester, England
7. Glasgow Royal Infirmary, Glasgow, Scotland
8. Ysbyty Ystrad Fawr, Aneurin Bevan University Health Board, Wales
9. North Bristol NHS Trust, Bristol, England
10. Cardiff University, Cardiff, Wales
11. University of Glasgow, Glasgow, Scotland
12. Department of Medicine for the Elderly, NHS Grampian, Aberdeen, Scotland

- Dr Eilidh Bruce- Academic Surgical Trainee and Honorary Research Fellow, Institute of Applied Health Science, University of Aberdeen, Aberdeen Scotland
- Dr Ben Carter- Senior Lecturer in Biostatistics and Health Informatics, Department of Biostatistics &Health Informatics, King’s College London, England
- Dr Terence J Quinn- Clinical Senior Lecturer, Cardiovascular & Medical Sciences. University of Glasgow, Glasgow, Scotland
- Dr Alessia Verduri- Consultant Respiratory Physician, University of Modena and Reggio Emilia - Hospital Policlinico Modena, Italy
- Oliver Pearson- Research assistant in Biostatistics and Health Informatics, Department of Biostatistics &Health Informatics, King’s College London, England
- Dr Arturo Vilches- Moraga- Consultant Geriatrician & Honorary Senior Lecturer, Salford Royal NHS Trust and, Salford
- Ms Angeline Price- Nurse Consultant, Manchester University, Manchester, England
- Dr Aine McGovern- Consultant Geriatrician, Glasgow Royal Infirmary, Glasgow, Scoltand
- Dr Louis Evans- Specialist Trainee Registrar in General Surgery, Ybsyty Gwynedd hospital Bangor, Wales
- Miss Kathryn McCarthy- Consultant Surgeon & Honorary Senior Lecturer, North Bristol NHS Trust, Bristol, England
- Dr Jonathan Hewitt- Clinical Senior Lecturer in Geriatric Medicine, Cardiff University, Cardiff, Wales
- Professor Susan J Moug- Consultant Surgeon and Honorary Professor, University of Glasgow
- Professor Phyo Kyaw Myint- Professor of Medicine of Old Age, Institute of Applied Health Science, University of Aberdeen, Aberdeen Scotland and Department of Medicine for the Elderly, NHS Grampian, Aberdeen, Scotland

**&COPE Study Collaborators:**

NHS Grampian & University of Aberdeen: Dr Alice Einarsson

Glasgow Royal Infirmary: Anna Fleck

Inverclyde Royal Infirmary: Dr Carly Bisset, Dr Ross Alexander.

Italy (University Hospital of Modena Policlinico): Professor Giovanni Guaraldi, MD

King’s College London: Caroline Murphy; Joanna Kelly; Dr Roxanna Short

North Bristol Trust: Dr Philip Braude, Tarik El Jichi Mutasem, Sandeep Singh, Dolcie Paxton, Will Harris, Dr James Hesford, Dr Mark Holloway, Dr Emma Mitchell, Dr Frances Rickard.

Royal Alexandra Hospital, Paisley: Norman Galbraith, Emma Bhatti, Jenny Edwards, and Siobhan Duffy, Dr Fenella Barlow-Pay

Salford Royal Infirmary: Lyndsey Pearce, Madeline Garcia, Shefali Sangani, Thomas Kneen, Thomas Lee

Ysbyty Yystad Fawr: Dr Charlotte Davey, Ms Sheila Jones, Kiah Lunstone, Alice Cavenagh, Charlotte Silver, Thomas Telford, Rebecca Simmons

Cardiff University: Mr Michael Stechman

**Correspondence to:**

Professor Phyo Myint

Institute of Applied Health Sciences, University of Aberdeen

Room 4:013, Polwart Building

School of Medicine and Dentistry

University of Aberdeen, Foresterhill

Aberdeen

AB25 2ZD

Tel: +44(0)1224 437841

Mail to: phyo.myint@abdn.ac.uk

**Study Registration Number:** IRAS project ID 281951

**Approval Numbers:** UK Health Research Authority (Reference: 20/HRA/1898)

Italy Ethics Committee of Policlinico Hospital Modena (Reference 369/2020/OSS/AOUMO)

**Protocol number:** SPON1815-20

**Abstract**

**Background:** In response to the COVID-19 pandemic, many countries mandated staying at home to reduce transmission. This study examined the association between living arrangements (house occupancy numbers) and outcomes in COVID-19.

**Methods:** Study population was drawn from the COPE Study, a multicentre cohort study. House occupancy was defined as: living alone; living with one other person; living with multiple other people; or living in a nursing/residential home. Outcomes were time from admission to mortality and discharge (Cox regression), and Day-28 mortality (logistic regression), analyses were adjusted for key comorbidities and covariates including admission: age; sex, smoking; heart failure; admission CRP; COPD; eGFR, frailty and others.

**Results:** 1584 patients were included from 13 hospitals across UK and Italy: 676 (42.7%) were female, 907 (57.3%) were male, median age was 74 years (range: 19-101). At 28 days, 502 (31.7%) had died. Median admission CRP was 67, 82, 79.5 and 83mg/L for those living alone, with someone else, in a house of multiple occupancy and in a nursing/residential home, respectively. Compared to living alone, living with anyone was associated with increased mortality: within a couple (aHR 1.39, 95%CI 1.09-1.77, p=0.007); living in a house of multiple occupancy (aHR=1.67, 95%CI 1.17-2.38, p=0.005); and living in a residential home (aHR=1.36, 95%CI 1.03-1.80, p=0.031).

**Conclusion:** For patients hospitalised with COVID-19, those living with one or more people had an increased association with mortality, they also exhibited higher CRP indicating increased disease severity suggesting they delayed seeking care.

**Key Words:** Covid-19; Coronavirus; Multiple House occupancy

**Introduction**

The Covid-19 pandemic has provided one of the greatest challenges known to public health. As of 15^th^ December 2020, there have been over 70 million cases and 1.6 million deaths attributed to the virus worldwide [1]. Transmission of the SARS- CoV- 2 virus is dependent on human interaction and behaviours, and this has been the single most important public health target across the globe [2]. In addition to handwashing [3] and the use of face- coverings [4], the public have been encouraged to physically distance and “Stay at Home” throughout. Furthermore, current UK law requires people to self- isolate if they or someone they live with are symptomatic or have a positive test [5]. More specifically, those infected with Covid-19 who live with another person or in a house of multiple occupancy are advised to self-isolate within their homes separate from other household members. This has been effective from a public health perspective in suppressing transmission of the virus.

However, it is not known whether outcomes in Covid-19 disease may be affected by social determinants such as living arrangements. Whilst isolation measures may reduce transmission, evidence is lacking with regard to how the environment in which people are isolating impacts on the severity of disease and outcomes for individuals who have contracted Covid-19. There has been no study to date that examines patient outcomes in relation to house occupancy. The primary aim of this study is to examine the association between living arrangements and in-hospital mortality in patients with Covid-19 infection, the secondary aim was to assess occupancy on time-to-discharge and disease severity estimated by serum CRP levels.

**Methods**

**Study design**

Data were collated as part of a European multicentre observational study: COPE (**C**OVID-19 in **O**lder **Pe**ople study) [6]. The protocol has been published elsewhere [7]. Ethical approval was obtained in the UK by the Health Research Authority (20/HRA/1898) and the Ethics Committee of Policlinico Hospital Modena, Italy (369/2020/OSS/AOUMO), respectively. This study has been reported following the STROBE statement [8]. A central MACRO database, hosted by King’s Clinical Trials Unit (KCTU), was used to collate the data centrally.

**Setting**

The study sites included an established network of twelve UK sites and one Italian site. The UK centres included: Aberdeen Royal Infirmary, Glasgow Royal Infirmary, Inverclyde Royal Hospital, Maidstone Hospital, Nevill Hall Hospital in Abergavenny, Royal Alexandra Hospital in Paisley, Royal Gwent Hospital in Newport, Southmead Hospital in Bristol, Salford Royal Hospital, University Hospital of Wales in Cardiff, Ysbyty Gwynedd in Bangor, and Ysbyty Ystrad Fawr in Caerphilly. The Italian centre was the University Hospital of Modena Policlinico.

**Participants**

Each site research team screened hospital admission lists daily between 27^th^ February and 10^th^ June 2020. The ethical approval was such that formal written consent from participants was not required as all data were routinely collected in hospital records.

*Inclusion/exclusion criteria*

The study included consecutive hospitalised adult patients aged 18 years or older with a confirmed diagnosis of COVID-19 admitted between 27^th^ February and 10^th^ June 2020; diagnostic criteria included laboratory confirmed SARS-CoV-2 positive swab or a clinical diagnosis of COVID-19; patients needed to be followed up at least for 28 days or until death.

**Outcome**

The primary outcome was length of time from admission to mortality. Secondary outcomes were the time-to-discharge, and Day 28 mortality. Patients who were discharged prior to Day 28 were censored at this point in the time to mortality analysis and assumed to be alive in the Day 28 mortality analysis, and those died were censored in the time to discharge analysis. Those alive and still in hospital with less than 28 days follow up were excluded from the Day 28 mortality analysis. For patients diagnosed with Covid-19 whilst as an inpatient the date of diagnosis was used rather than date of admission to hospital. Other prespecified outcomes included in the COPE study were the effect of drug classes, nosocomial infection and frailty, however these are not analysed here and have been reported in previous publications [6,9-11].

**Primary exposure**

Home occupancy was categorised as: living alone; living with one other person; living in a house of multiple occupancy (not in a residential or nursing home); living in a residential or nursing care home.

**Covariates**

Demographic and clinical characteristics recorded at admission were: age, sex, smoking status (never, previous, or current), C-reactive protein (CRP) as a marker of disease severity (REF Stringer et al, under revision with Int J Epidemiol), estimated glomerular filtration rate (eGFR), previous history of coronary artery disease (CAD), diabetes mellitus, chronic obstructive pulmonary disease (COPD), hypertension (no, yes not on treatment, and yes on treatment), albumin, heart failure, and frailty (using the Clinical Frailty Scale; CFS).

**Data Analysis**

Admission demographic and clinical characteristics were presented by Day-28 mortality, to describe the included participant characteristics.

Time to mortality (primary outcome) and length of stay (secondary outcome) were analysed with mixed-effects multivariable Cox’s proportional baseline hazards regression models. The analyses were fitted with a random effect to account for hospital variation (26), and adjusted for the base model of: patient age group; sex; smoking status; CRP; diabetes; hypertension; eGFR; COPD; coronary artery disease (CAD); albumin; heart failure; and frailty (CFS; 1-3 versus 4-5 and 6-9). The adjusted hazard ratios (aHR) were estimated with associated 95% confidence intervals (95% CI). The baseline proportionality assumption was tested visually with log-log residuals. Each time to event analysis was reported with a Kaplan Meier survival plot.

The secondary outcome of Day-28 mortality was analysed using a mixed-effects multivariable logistic model, fitting each hospital as a random intercept effect, and adjusted with covariates consistent with the primary outcome. The adjusted odds ratio (aOR) were presented with associated 95% CI. Missing data were explored for patterns of missingness. Analysis was carried out using Stata/MP version 16.0.

Deprivation, using the Index of Multiple Deprivation (IMD 1-3 versus 4-7 and 8-10) was additionally fitted to the primary analysis of the mixed-effects multivariable Cox PH analysis as a sensitivity analysis.

**Results**

The study included 1671 patients from 13 hospitals; however, 87 (5.2%) did not have 28 days follow up (or mortality) and were excluded. Of the 1584 included patients, 676 (42.7%) were female, 907 (57.3%) were male, and median age was 74 years (IQR: 61.5-83; range: 19-101), and 1417 (89.5%) were white (Table 1). With regard to co-morbidites, 38.5% were taking medication for hypertension, and 60.0% had low albumin. In hospital mortality in patients with at least 28 day follow up (or who died) was 31.7% (502/1584). There were 433 patients who lived alone and 32.1% died, compared to 29.4% who died and lived with one other. Of 189 patients that lived in house of multiple occupancy outside of a nursing or residential home 25.4% died, compared to 47.0% who lived in a nursing or residential home. There were 141 cases (8.9%) where housing occupancy was not reported.

The Kaplan Meier overall survival plot demonstrates the association between residential or nursing care and mortality (Figure 1). However, little differences are suggested from the other three house occupancy groups. The demographic and clinical characteristics of the house occupancy distribution shows that patients who live alone were more likely to be older, than those living in a couple of in a house of multiple occupancy (Supplementary Table 1). The admission CRP for those living alone was median=67 mg/L (28-130 IQR), compared to those living as a couple, median=82 mg/L (34-155 IQR), or living in a house of multiple occupancy median=79.5 mg/L (45-150 IQR) or living in a nursing home median CRP=83 mg/L (35-138 IQR).

**Data Analysis**

Within the crude mixed-effects Cox regression analysis, there was an association between living alone versus living in a care home, and mortality HR=1.60 (95% CI 1.24-2.05; p<0.001), the full set of crude analyses can be found in Table 2.

Within the adjusted multivariable Cox regression, house occupancy was associated with mortality. Compared to living alone, co-habiting was associated with increased mortality such as: within in a couple (aHR1.39, 95% CI 1.09-1.77, p=0.007); living in a house of multiple occupancy (aHR=1.67, 95% CI 1.17-2.38, p=0.005); and living in a nursing or residential home (aHR=1.36, 95% CI 1.03-1.80, p=0.031). Other important covariates in the associated with mortality in the adjusted analysis were: age; CRP; eGFR, albumin, and COPD, full details can be found in Table 2.

In the secondary outcome 28 Day mortality, occupancy was associated with mortality for patients in a nursing or residential home OR=1.88 (95% CI 1.34-2.64, p<0.001), Table 3. Other covariates associated with morality in this outcome were age, smoking, CAD, CRP, eGFR, albumin, COPD and CHF, Table 3. In the secondary outcome of time to discharge there was a crude association between having someone at home and increased discharge (compared to living alone), living as a couple HR=1.31 95% CI 1.11-1.55, p=0.002; and living with multiple others HR=1.56 95% CI 1.25-1.95, p<0.001), Supplementary Table 2. In the adjusted multivariable analysis there was an association between living alone verses in a residential/nursing home, and time to discharge (aHR=1.41 95% CI 1.08-1.84, p=0.012).

After additionally adjusting for deprivation using the Index of Multiple Deprivation (IMD 1-3, versus 4-7, and 8-10), there was no change in the effect of occupancy, and both the magnitude and significance of each finding were maintained.

**Discussion**

Our study reports the novel association between home living arrangements and outcomes in hospitalized patients with Covid-19. In this large prospective study, we have provided further evidence on the association of residential/ care home living and increased mortality from the disease. However, we also present the novel finding that multiple house occupancy (i.e. living with either one or multiple other people) is associated with increased mortality in Covid-19 infection in community settings.

The association between nursing home residence and mortality in Covid-19 is well described. Although less than 0.5% of the total population of the United States live in nursing homes, nursing home residents have accounted for around 25% of the documented deaths in Covid-19 [12]. A number of American states published their individual data during the early months of the pandemic. This has shown that deaths in long term care facilities have accounted for over 50% of all Covid-19 deaths in the states of Delaware, Massachusetts, Oregon, Pennsylvania, Colorado and Utah [13].

Our findings may be explained by the effect of public health messages delaying individual patients presenting to hospital. If incapacitated by the virus, those living alone may be less able to cope with their symptoms and the difficulties caused by self-isolation, therefore more likely to exhibit health seeking behaviour and thus present to hospital earlier with less severe disease. This could explain the lower admission CRP levels and subsequent lower mortality risk seen in this patient group. Conversely, those living with others may be encouraged to remain at home for longer despite a worsening clinical state and are possible to do so by support of other household members.

There are a number of plausible theories that may explain the observed associations we found. A large prospective study has shown an independent relationship between high viral load and mortality, adjusted for age, sex and common comorbidities [14]. If this evidence is considered in the context of living arrangements, those who live with others are at risk of contracting the virus from another member of their household. In the lockdown climate of the early pandemic (when COPE was conducted), the public health message to stay at home rendered many households isolating together, exposing other members of the household to the virus if one were to contract it. It is therefore possible that those living in houses of multiple occupancy have increased viral load exposure and resultant increased mortality outcomes from Covid-19. Multiple house occupancy was also seen to be associated with raised CRP at admission, which can be interpreted as a proxy for increased disease severity [15]. This is clinically relevant to public health policy makers. The message should be very clear, that strict self-isolation for Covid-19 positive people within houses of multiple occupancy is vital in order to reduce virus exposure to other members of the household. It may be advisable to consider living alone as a vulnerable person to reduce risk of developing severe disease.

Indeed, these theories are supported by knowledge of health seeking behaviours and how they have been shaped by the Covid-19 pandemic. Fear and threat are central to the emotional responses felt by many during a pandemic [16]. Negative framing of the pandemic from the media’s perspective often fuels this, for example by reporting the number of cases and deaths rather than the data on those who recover. Those who are not mathematically skilled with knowledge regarding probabilities and risk are particularly susceptible to fear as a result of negative framing [17]. Fear and strict adherence to public health instruction to stay at home may have encouraged people to keep their unwell family members at home until they deteriorate to extremis. This could account for higher admission CRP and mortality risk in those from houses of multiple occupancy, observed in this study.

This was an observational study and is therefore is subject to the intrinsic limitations associated with all studies of this nature. The prospective nature of the data collection reduces the possibility of reverse causality, and the collection of unselected and consecutive data for all patients admitted with Covid-19 acts to reduce selection bias. The study was limited to those hospitalized, it also does not capture data from patients who remained in the community or patients who were discharged from (or died) in the Emergency Department. The impact of confounding variables has also been addressed by statistical adjustment for variables including age, co-morbidities and admission CRP. Patients with nosocomial Covid-19 infection (infection that is acquired by a patient who was admitted for another reason) were a small percentage of the study population, and are unlikely to impact on the results as they have been found to have a lower mortality rate than patients with community acquired infection [11]. We did not collect the duration of symptoms prior to admission and also did not collect the isolation arrangements between household members since onset of the symptoms, but this is of less relevance as it is well known that infectivity begins prior to symptom onset. We did not contact trace to household members and thus unable to uncertain to an extent that findings are contributed by infection from other household member who did not require hospital admission but contributed to potentially higher viral load depicted by higher CRP levels among those from homes with multiple occupancy. Information regarding dependency was not collected, and therefore it is also possible that these findings are confounded by the possibility that those people in houses of multiple occupancy are dependent and therefore at risk of worse outcomes. The analyses did not adjust for ethnicity because the majority of the sample was white (89.5%), therefore an analysis of ethnicity would be underpowered. We did not collect the detailed information on the treatment received by patients, but at the time of the study, the understanding of treatment of COVID-19 was sparse, and all centres are likely to have had similar approaches in management.

We found these patients from multiple occupancy households to have increased levels of CRP at time of presentation, a proxy marker for disease severity. This is novel information into the impact of living arrangements and outcomes in Covid-19 disease, during a pandemic where there has been much uncertainty. Public health measures, although effective at preventing overall disease transmission, should further highlight the importance of self-isolation within a household to reduce the possible effect of increased viral load. Messages should also encourage patients who are clinically deteriorating at home to present to hospital for appropriate treatment. We believe that the implications of these findings are relevant not only to the second wave of the current Covid-19 pandemic, but to future public health crises of highly contagious diseases.

**Funding**

None.

**Conflict of Interest**

None for all authors.

**Contribution of authors**

JH and KM are PIs of COPE study. All authors contributed to data collection, interpretation of results and writing of the manuscript. BC is the guarantor.

**Acknowledgement**

We acknowledge the dedication, commitment, and sacrifice of the staff from participating centres across UK and Italy, two amongst the most severely affected countries in Europe. We gratefully acknowledge the contribution of our collaborators, National Institute of Health Research (NIHR) Health Research Authority (HRA) in the UK and Ethics Committee of Policlinico Hospital Modena, which provided rapid approval of COPE study and respective Institutions’ Research and Development Offices and Caldicott Guardians for their assistance and guidance. We also thank COPE Study Sponsor, Cardiff University, Wales, UK.

**Key Points**

- Multiple house occupancy (i.e. living with either one or multiple other people) is associated with increased mortality in Covid-19 infection in community settings.
- Patients from multiple occupancy households to have increased levels of CRP at time of presentation, a proxy marker for disease severity.
- Mortality risk in COVID-19 is multi-factorial and should be considered in the context of environmental circumstances as well as clinical and demographic variables.

**References**

1. World Health Organisation. WHO Covid-19 Weekly Epidemiological Update. [updated 15 December 2020; cited 20 December 2020]. Available from: https://www.who.int/publications/m/item/weekly-epidemiological-update---15-december-2020.
2. M Komaroff, A A Belhouchet, Public Health Policies and Global COVID-19 Outbreak, European Journal of Public Health, Volume 30, Issue Supplement_5, September 2020, ckaa165.428, <https://doi.org/10.1093/eurpub/ckaa165.428>
3. Alzyood M, Jackson D, Aveyard H, Brooke J. COVID-19 reinforces the importance of handwashing. J Clin Nurs. 2020;29(15-16):2760-2761. doi:10.1111/jocn.15313
4. Chu DK, Akl EA, Duda S, et al. Physical distancing, face masks, and eye protection to prevent person-to-person transmission of SARS-CoV-2 and COVID-19: a systematic review and meta-analysis. Lancet. 2020;395(10242):1973-1987. doi:10.1016/S0140-6736(20)31142-9
5. National Health Service. Coronavirus (COVID-1) Self-isolation and treating coronavirus symptoms. [cited 30 November 2020]. Available from: <https://www.nhs.uk/conditions/coronavirus-covid-19/self-isolation-and-treatment/>
6. Hewitt J, Carter B, Vilches-Moraga A, Quinn TJ, Braude P, Verduri A, Pearce L, Stechman M, Short R, Price A, Collins J, Bruce E, Einarsson A, Rickard F, Mitchell E, Holloway M, Hesford J, Barlow-Pay F, Clini E, Myint PK, Moug SJ and McCarthy K on behalf of the COPE Study Collaborators* The COPE study: COVID-19 in Older PEople – the influence of frailty on survival. A multi-centre, European observational study. Lancet Public Health 2020; 5(8):e444-e451.
7. Price, A., Barlow-Pay, F., Duffy, S., Pearce, L., Vilches-Moraga, A., Moug, S., Quinn, T., Stechman, M., Braude, P., Mitchell, E., Myint, P. K., Verduri, A., McCarthy, K., Carter, B., Hewitt, J., & COPE Study Collaborators (2020). Study protocol for the COPE study: COVID-19 in Older PEople: the influence of frailty and multimorbidity on survival. A multicentre, European observational study. *BMJ open*, *10*(9), e040569. https://doi.org/10.1136/bmjopen-2020-040569
8. von Elm E, Altman DG, Egger M, Pocock SJ, Gøtzsche PC, Vandenbroucke JP; STROBE Initiative.
   **The Strengthening the Reporting of Observational Studies in Epidemiology (STROBE)statement: guidelines for reporting observational studies.**
   [Lancet. 2007 Oct 20](http://www.thelancet.com/journals/lancet/article/PIIS0140-6736(07)61602-X/fulltext#article_upsell);370(9596):1453-7. PMID: 18064739
9. Bruce E, Barlow-Pay F, Short R, Vilches-Moraga A, Price A, McGovern A, Braude P, Stechman MJ, Moug S, McCarthy K, Hewitt J, Carter B, Myint PK. Prior Routine Use of Non-Steroidal Anti-Inflammatory Drugs (NSAIDs) and Important Outcomes in Hospitalised Patients with COVID-19. J Clin Med. 2020 Aug 10;9(8):2586. doi: 10.3390/jcm9082586. PMID: 32785086; PMCID: PMC7465199.
10. Braude P, Carter B, Short R, Vilches-Moraga A, Verduri A, Pearce L, Price A, Quinn TJ, Stechman M, Collins J, Bruce E, Einarsson A, Rickard F, Mitchell E, Holloway M, Hesford J, Barlow-Pay F, Clini E, Myint PK, Moug S, McCarthy K, Hewitt J. The influence of ACE inhibitors and ARBs on hospital length of stay and survival in people with COVID-19. Int J Cardiol Heart Vasc. 2020 Dec;31:100660. doi: 10.1016/j.ijcha.2020.100660. Epub 2020 Oct 15. PMID: 33083516; PMCID: PMC7561344.
11. Carter B, Collins JT, Barlow-Pay F, Rickard F, Bruce E, Verduri A, Quinn TJ, Mitchell E, Price A, Vilches-Moraga A, Stechman MJ, Short R, Einarsson A, Braude P, Moug S, Myint PK, Hewitt J, Pearce L, McCarthy K; COPE Study Collaborators. Nosocomial COVID-19 infection: examining the risk of mortality. The COPE-Nosocomial Study (COVID in Older PEople). J Hosp Infect. 2020 Oct;106(2):376-384. doi: 10.1016/j.jhin.2020.07.013. Epub 2020 Jul 21. PMID: 32702463; PMCID: PMC7372282.
12. Grabowski DC, Mor V. Nursing Home Care in Crisis in the Wake of COVID-19. *JAMA.* 2020;324(1):23–24. doi:10.1001/jama.2020.8524
13. Chidambaram  P﻿. Kaiser Family Foundation Issue Brief: state reporting of cases and deaths due to COVID-19 in long-term care facilities. Accessed April 26, 2020. <https://www.kff.org/medicaid/issue-brief/state-reporting-of-cases-and-deaths-due-to-covid-19-in-long-term-care-facilities/>
14. Pujadas E, Chaudhry F, McBride R, Richter F, Zhao S, Wajnberg A, Nadkarni G, Glicksberg BS, Houldsworth J, Cordon-Cardo C. SARS-CoV-2 viral load predicts COVID-19 mortality. Lancet Respir Med. 2020 Sep;8(9):e70. doi: 10.1016/S2213-2600(20)30354-4. Epub 2020 Aug 6. PMID: 32771081.
15. Stringer D, Braude P, Myint P, Evans L, Collins J, Verduri A, Quinn T, Vilches-Moraga A, Stechman M, Pearce L, Moug S, McCarthy K, Hewitt J, Carter B, COPE Study Collaborators, The role of C-reactive protein as a prognostic marker in COVID-19, International Journal of Epidemiology, 2021; dyab012, <https://doi.org/10.1093/ije/dyab012>
16. Bavel, J.J.V., Baicker, K., Boggio, P.S. *et al.* Using social and behavioural science to support COVID-19 pandemic response. *Nat Hum Behav* **4,**460–471 (2020). https://doi.org/10.1038/s41562-020-0884-z
17. Peters, E. et al. Numeracy and decision making. *Psychol. Sci.* **17**, 407–413 (2006).

**Table 1. Demographics and comorbidities for the included patients**

|  | **Mortality at Day 28** | |  |
| --- | --- | --- | --- |
|  | **Alive** | **Dead** | **Total** |
|  | **N = 1,082** | **N = 502** | **N = 1584** |
| **Sex** |  |  |  |
| Female | 468 (69.2) | 208 (30.8) | 676 (42.7) |
| Male | 613 (67.6) | 294 (32.4) | 907 (57.3) |
| Missing | 1 | 0 | 1 |
| **Age** |  |  |  |
| ≤64 | 420 (87.3) | 61 (12.7) | 481 (30.4) |
| 65–74 | 221 (70.8) | 91 (29.2) | 312 (19.7) |
| 75–84 | 257 (57.1) | 193 (42.9) | 450 (28.4) |
| 85+ | 184 (54.0) | 157 (46.0) | 341 (21.5) |
| **Ethnicity** |  |  |  |
| White | 957 (67.5) | 460 (32.5) | 1417 (89.5) |
| Asian/Asian British | 32 (68.1) | 15 (31.9) | 47 (3.0) |
| Black/Black British | 17 (77.3) | 5 (22.7) | 22 (1.4) |
| Chinese | 3 (75.0) | 1 (25.0) | 4 (0.3) |
| Mixed | 1 (100.0) | 0 (0.0) | 1 (0.1) |
| Other | 11 (91.7) | 1 (8.3) | 12 (0.8) |
| Missing | 61 | 20 | 81 |
| **Living arrangement** |  |  |  |
| Lives alone | 294 (67.9) | 139 (32.1) | 433 (27.3) |
| Lives with 1 other | 425 (70.6) | 177 (29.4) | 602 (38) |
| Lives with multiple others (not in a residential or nursing home) | 141 (74.6) | 48 (25.4) | 189 (11.9) |
| Lives with multiple others (in a residential or nursing home) | 116 (53.0) | 103 (47.0) | 219 (13.8) |
| Missing | 106 | 35 | 141 |
| **Smoking** |  |  |  |
| Never smoked | 587 (71.2) | 237 (28.8) | 824 (52.0) |
| Ex-smoker | 381 (63.0) | 224 (37.0) | 605 (38.2) |
| Current smoker | 85 (75.9) | 27 (24.1) | 112 (7.1) |
| Missing | 29 | 14 | 43 |
| **Diabetes** |  |  |  |
| No | 812 (69.7) | 353 (30.3) | 1165 (73.6) |
| Yes | 268 (64.4) | 148 (35.6) | 416 (26.3) |
| Missing | 2 | 1 | 3 |
| **Coronary Artery Disease** |  |  |  |
| No | 896 (71.6) | 356 (28.4) | 1252 (79.0) |
| Yes | 185 (56.1) | 145 (43.9) | 330 (20.8) |
| Missing | 1 | 1 | 2 |
| **Hypertension** |  |  |  |
| No | 560 (70.1) | 239 (29.9) | 799 (50.4) |
| Yes | 112 (64.7) | 61 (35.3) | 173 (10.9) |
| Yes (on treatment) | 409 (67.2) | 200 (32.8) | 609 (38.5) |
| Missing | 1 | 2 | 3 |
| **CRP** |  |  |  |
| <40 | 380 (82.3) | 82 (17.8) | 462 (29.2) |
| ≥40 | 689 (62.9) | 406 (37.1) | 1095 (69.1) |
| Missing | 13 | 14 | 27 |
| **eGFR** |  |  |  |
| ≥60 | 745 (75.7) | 239 (24.3) | 984 (62.1) |
| 45–59 | 120 (61.5) | 75 (38.5) | 195 (12.3) |
| 30–44 | 105 (54.4) | 88 (45.6) | 193 (12.2) |
| <30 | 91 (53.9) | 78 (46.2) | 169 (10.7) |
| Missing | 21 | 22 | 43 |
| **Albumin** |  |  |  |
| ≥35 | 425 (76.2) | 133 (23.8) | 558 (35.2) |
| <35 | 606 (63.7) | 345 (36.3) | 951 (60.0) |
| Missing | 51 | 24 | 75 |
| **COPD** |  |  |  |
| No | 914 (70.1) | 390 (29.9) | 1304 (82.3) |
| Yes | 116 (57.7) | 85 (42.3) | 201 (12.7) |
| Missing | 52 | 27 | 79 |
| **Heart Failure** |  |  |  |
| No | 940 (70.4) | 396 (29.6) | 1336 (84.3) |
| Yes | 90 (54.2) | 76 (45.8) | 166 (10.5) |
| Missing | 52 | 30 | 82 |
| **Clinical Frailty Scale** |  |  |  |
| 1-3 | 468 (83.4) | 93 (16.6) | 561 (35.4) |
| 4-5 | 257 (68.2) | 120 (31.8) | 377 (23.8) |
| 6-9 | 351 (55.3) | 284 (44.7) | 635 (40.0) |
| Missing | 6 | 5 | 11 |

**Table 2. Crude and Multivariable Cox proportional hazards regression presenting crude, and adjusted^&^ Hazard Ratio (HR) analysis of the time to mortality**

|  | **Crude HR (95% CI)** | **p value** | **Adjusted HR^&^ (95% CI)** | **p value** |
| --- | --- | --- | --- | --- |
| **Sex** |  |  |  |  |
| Female | Reference |  | Reference |  |
| Male | 1.05 (0.88-1.25) | 0.579 | 1.21 (0.99-1.48) | 0.060 |
| **Age** |  |  |  |  |
| <65 | Reference |  | Reference |  |
| 65–74 | 2.33 (1.70-3.20) | <0.001 | 1.85 (1.47-2.68) | 0.001 |
| 75–84 | 3.68 (2.78-4.88) | <0.001 | 2.93 (2.04-4.21) | <0.001 |
| 85+ | 3.85 (2.88-5.14) | <0.001 | 3.08 (2.10-4.54) | <0.001 |
| **Smoking** |  |  |  |  |
| Never smoked | Reference |  | Reference |  |
| Ex-smoker | 1.26 (1.05-1.50) | 0.012 | 1.00 (0.82-1.24) | 0.867 |
| Current smoker | 0.76 (0.51-1.12) | 0.169 | 0.93 (0.61-1.41) | 0.725 |
| **Diabetes** |  |  |  |  |
| No | Reference |  | Reference |  |
| Yes | 1.11 (0.92-1.34) | 0.288 | 1.05 (0.85-1.30) | 0.630 |
| **Coronary Artery Disease** |  |  |  |  |
| No | Reference |  | Reference |  |
| Yes | 1.57 (1.29-1.90) | <0.001 | 1.13 (0.90-1.41) | 0.307 |
| **Hypertension** |  |  |  |  |
| No | Reference |  | Reference |  |
| Yes | 1.12 (0.85-1.49) | 0.412 | 0.90 (0.66-1.24) | 0.522 |
| Yes (on treatment) | 1.15 (0.95-1.38) | 0.150 | 0.91 (0.74-1.13) | 0.414 |
| **CRP** |  |  |  |  |
| <40 | Reference |  | Reference |  |
| ≥40 | 1.92 (1.55-2.38) | <0.001 | 2.04 (1.61-2.60) | <0.001 |
| **eGFR** |  |  |  |  |
| ≥60 | Reference |  | Reference |  |
| 45–59 | 1.77 (1.37-2.29) | <0.001 | 1.48 (1.12-1.95) | 0.006 |
| 30–44 | 2.06 (1.62-2.62) | <0.001 | 1.48 (1.13-1.95) | 0.004 |
| <30 | 1.83 (1.42-2.37) | <0.001 | 1.50 (1.13-1.97) | 0.004 |
| **Albumin** |  |  |  |  |
| ≥35 | Reference |  | Reference |  |
| <35 | 1.45 (1.17-1.78) | 0.001 | 1.34 (1.06-1.70) | 0.014 |
| **COPD** |  |  |  |  |
| No | Reference |  | Reference |  |
| Yes | 1.58 (1.26-1.99) | <0.001 | 1.31 (1.01-1.70) | 0.039 |
| **Heart Failure** |  |  |  |  |
| No | Reference |  | Reference |  |
| Yes | 1.66 (1.30-2.12) | <0.001 | 1.09 (0.83-1.44) | 0.528 |
| **Clinical Frailty Scale** |  |  |  |  |
| 1-3 | Reference |  | Reference |  |
| 4-5 | 1.70 (1.30-2.21) | <0.001 | 1.18 (0.86-1.64) | 0.307 |
| 6-9 | 2.45 (1.94-3.11) | <0.001 | 1.49 (1.09-2.05) | 0.013 |
| **Living arrangement** |  |  |  |  |
| Lives alone | Reference |  | Reference |  |
| Lives with 1 other | 1.05 (0.84-1.31) | 0.691 | 1.39 (1.09-1.77) | 0.007 |
| Lives with multiple others (not in a residential or nursing home) | 0.97 (0.69-1.34) | 0.836 | 1.67 (1.17-2.38) | 0.005 |
| Lives with multiple others (in a residential or nursing home) | 1.60 (1.24-2.05) | <0.001 | 1.36 (1.03-1.80) | 0.031 |

^&^Multivariable analysis was adjusted by: sex; age; smoking; diabetes; coronary artery disease; hypertension; CRP; eGFR; albumin, COPD, heart failure and Clinical frailty Scale.

Note: the number of observations excluded due to missing data was 227

**Table 3. Crude and Multivariable Logistic regression presenting crude, and adjusted multivariable^&^ odds ratio (OR) for mortality at Day 28**

|  | **Crude OR (95% CI)** | **p value** | **Adjusted OR^&^ (95% CI)** | **p value** |
| --- | --- | --- | --- | --- |
| **Sex** |  |  |  |  |
| Female | Reference |  | Reference |  |
| Male | 1.12 (0.90-1.40) | 0.310 | 1.44 (1.09-1.90) | 0.010 |
| **Age** |  |  |  |  |
| ≤64 | Reference |  | Reference |  |
| 65–74 | 3.28 (2.26-4.76) | <0.001 | 2.17 (1.38-3.39) | 0.001 |
| 75–84 | 6.26 (4.43-8.83) | <0.001 | 4.20 (2.69-6.55) | <0.001 |
| 85+ | 7.53 (5.22-10.87) | <0.001 | 4.96 (3.03-8.12) | <0.001 |
| **Smoking** |  |  |  |  |
| Never smoked | Reference |  | Reference |  |
| Ex-smoker | 1.43 (1.14-1.79) | 0.002 | 1.06 (0.79-1.42) | 0.690 |
| Current smoker | 0.72 (0.45-1.15) | 0.174 | 0.99 (0.57-1.75) | 0.985 |
| **Diabetes** |  |  |  |  |
| No | Reference |  | Reference |  |
| Yes | 1.20 (0.94-1.53) | 0.144 | 1.06 (0.78-1.43) | 0.725 |
| **Coronary Artery Disease** |  |  |  |  |
| No | Reference |  | Reference |  |
| Yes | 1.97 (1.52-2.55) | <0.001 | 1.17 (0.84-1.61) | 0.349 |
| **Hypertension** |  |  |  |  |
| No | Reference |  | Reference |  |
| Yes | 1.21 (0.84-1.73) | 0.306 | 0.80 (0.51-1.25) | 0.322 |
| Yes (on treatment) | 1.24 (0.98-1.57) | 0.075 | 0.94 (0.69-1.26) | 0.661 |
| **CRP** |  |  |  |  |
| <40 | Reference |  | Reference |  |
| ≥40 | 2.50 (1.92-3.25) | <0.001 | 2.60 (1.90-3.56) | <0.001 |
| **eGFR** |  |  |  |  |
| ≥60 | Reference |  | Reference |  |
| 45–59 | 2.05 (1.47-2.87) | <0.001 | 1.38 (0.94-2.03) | 0.098 |
| 30–44 | 2.62 (1.89-3.64) | <0.001 | 1.41 (0.95-2.10) | 0.087 |
| <30 | 2.46 (1.74-3.47) | <0.001 | 1.81 (1.19-2.73) | 0.005 |
| **Albumin** |  |  |  |  |
| ≥35 | Reference |  | Reference |  |
| <35 | 2.35 (1.79-3.08) | <0.001 | 1.88 (1.36-2.60) | <0.001 |
| **COPD** |  |  |  |  |
| No | Reference |  | Reference |  |
| Yes | 1.72 (1.26-2.35) | 0.001 | 1.32 (0.90-1.95) | 0.155 |
| **Heart Failure** |  |  |  |  |
| No | Reference |  | Reference |  |
| Yes | 2.07 (1.47-2.90) | <0.001 | 1.08 (0.72-1.64) | 0.698 |
| **Frailty** |  |  |  |  |
| 1-3 | Reference |  | Reference |  |
| 4-5 | 2.37 (1.72-3.26) | <0.001 | 1.29 (0.86-1.94) | 0.226 |
| 6-9 | 4.46 (3.32-5.99) | <0.001 | 2.26 (1.51-3.41) | <0.001 |
| **Living arrangement** |  |  |  |  |
| Lives alone | Reference |  | Reference |  |
| Lives with 1 other | 0.91 (0.69-1.20) | 0.505 | 1.34 (0.97-1.87) | 0.077 |
| Lives with multiple others (not in a residential or nursing home) | 0.70 (0.47-1.05) | 0.085 | 1.46 (0.90-2.35) | 0.121 |
| Lives with multiple others (in a residential or nursing home) | 1.88 (1.34-2.64) | <0.001 | 1.37 (0.91-2.06) | 0.134 |

^&^Multivariable analysis was adjusted by: age; sex; smoking; diabetes; coronary artery disease; hypertension; CRP; eGFR; albumin, COPD, heart failure and frailty.

Note: the number of observations excluded due to missing data was 227
