# Supplementary material for: Multiple house occupancy is associated with mortality in hospitalized patients with COVID-19
Source: Eur J Public Health. 2021 May 17;32(1):133–9. doi: 10.1093/eurpub/ckab085 (PMC8247274; doi:10.1093/eurpub/ckab085)
Supplement: ckab085_Supplementary_Data [file ckab085_supplementary_data.zip › ejph-2021-01-om-0093-File004.docx]

**Supplementary Table 1. Demographics and comorbidities against living arrangement**

|  |  |  | **Lives with multiple people** | |  |  |
| --- | --- | --- | --- | --- | --- | --- |
|  | **Lives alone** | **Lives with 1 other** | **Not in a residential or nursing home** | **In a residential or nursing home** | **Missing** | **Total** |
|  | **N = 433** | **N = 602** | **N = 189** | **N = 219** | **N = 141** | **N = 1584** |
| **Sex** |  |  |  |  |  |  |
| Female | 220 (32.5) | 216 (32.0) | 66 (9.8) | 119 (17.6) | 55 (8.1) | 676 (42.7) |
| Male | 213 (23.5) | 386 (42.6) | 123 (13.6) | 99 (10.9) | 86 (9.5) | 907 (57.3) |
| Missing | 0 | 0 | 0 | 1 | 0 | 1 |
| **Age** |  |  |  |  |  |  |
| ≤64 | 81 (16.8) | 203 (42.2) | 113 (23.5) | 12 (2.5) | 72 (15.0) | 481 (30.4) |
| 65–74 | 66 (21.2) | 150 (48.1) | 32 (10.3) | 33 (10.6) | 31 (9.9) | 312 (19.7) |
| 75–84 | 140 (31.1) | 177 (39.3) | 25 (5.6) | 86 (19.1) | 22 (4.9) | 450 (28.4) |
| 85+ | 146 (42.8) | 72 (21.1) | 19 (5.6) | 88 (25.8) | 16 (4.7) | 341 (21.5) |
| **Smoking** |  |  |  |  |  |  |
| Never smokers | 209 (25.4) | 313 (38.0) | 109 (13.2) | 123 (14.9) | 70 (8.5) | 824 (52.0) |
| Ex-smokers | 177 (29.3) | 234 (38.7) | 66 (10.9) | 75 (12.4) | 53 (8.8) | 605 (38.2) |
| Current smokers | 41 (36.6) | 38 (33.9) | 12 (10.7) | 9 (8.0) | 12 (10.7) | 112 (7.1) |
| Missing | 6 | 17 | 2 | 12 | 6 | 43 |
| **Diabetes** |  |  |  |  |  |  |
| No | 302 (25.9) | 448 (38.5) | 139 (11.9) | 162 (13.9) | 114 (9.8) | 1165 (73.6) |
| Yes | 131 (31.5) | 153 (36.8) | 50 (12.0) | 55 (13.2) | 27 (6.5) | 416 (26.3) |
| Missing | 0 | 1 | 0 | 2 | 0 | 3 |
| **Coronary Artery Disease** |  |  |  |  |  |  |
| No | 328 (26.2) | 469 (37.5) | 168 (13.4) | 163 (13.0) | 124 (9.9) | 1252 (79.0) |
| Yes | 105 (31.8) | 132 (40.0) | 21 (6.4) | 55 (16.7) | 17 (5.2) | 330 (20.8) |
| Missing | 0 | 1 | 0 | 1 | 0 | 2 |
| **Hypertension** |  |  |  |  |  |  |
| No | 216 (27.0) | 280 (35.0) | 111 (13.9) | 122 (15.3) | 70 (8.8) | 799 (50.4) |
| Yes | 52 (30.1) | 50 (28.9) | 16 (9.3) | 34 (19.7) | 21 (12.1) | 173 (10.9) |
| Yes (and on treatment) | 165 (27.1) | 272 (44.7) | 61 (10.0) | 63 (10.3) | 48 (7.9) | 609 (38.5) |
| Missing | 0 | 0 | 1 | 0 | 2 | 3 |
| **CRP** |  |  |  |  |  |  |
| <40 | 152 (32.9) | 172 (37.2) | 44 (9.5) | 62 (13.4) | 32 (6.9) | 462 (29.2) |
| ≥40 | 279 (25.5) | 419 (38.3) | 142 (13.0) | 153 (14.0) | 102 (9.3) | 1095 (69.1) |
| Missing | 2 | 11 | 3 | 4 | 7 | 27 |
| **eGFR** |  |  |  |  |  |  |
| ≥60 | 237 (24.1) | 403 (41.0) | 137 (13.9) | 110 (11.2) | 97 (9.9) | 984 (62.1) |
| 45-59 | 71 (36.4) | 69 (35.4) | 17 (8.7) | 31 (15.9) | 7 (3.6) | 195 (12.3) |
| 30-44 | 73 (37.8) | 59 (30.6) | 17 (8.8) | 34 (17.6) | 10 (5.2) | 193 (12.2) |
| <30 | 47 (27.8) | 63 (37.3) | 14 (8.3) | 37 (21.9) | 8 (4.7) | 169 (10.7) |
| Missing | 5 | 8 | 4 | 7 | 19 | 43 |
| **Albumin** |  |  |  |  |  |  |
| ≥35 | 138 (24.7) | 210 (37.6) | 83 (14.9) | 71 (12.7) | 56 (10.0) | 558 (35.2) |
| <35 | 278 (29.2) | 369 (38.8) | 97 (10.2) | 131 (13.8) | 76 (8.0) | 951 (60.0) |
| Missing | 17 | 23 | 9 | 17 | 9 | 75 |
| **COPD** |  |  |  |  |  |  |
| No | 345 (26.5) | 490 (37.6) | 165 (12.7) | 180 (13.8) | 124 (9.5) | 1304 (82.3) |
| Yes | 71 (35.3) | 72 (35.8) | 19 (9.5) | 26 (12.9) | 13 (6.5) | 201 (12.7) |
| Missing | 17 | 40 | 5 | 13 | 4 | 79 |
| **Heart Failure** |  |  |  |  |  |  |
| No | 350 (26.2) | 502 (37.6) | 172 (12.9) | 185 (13.9) | 127 (9.5) | 1336 (84.3) |
| Yes | 66 (39.8) | 59 (35.5) | 13 (7.8) | 21 (12.7) | 7 (4.2) | 166 (10.5) |
| Missing | 17 | 41 | 4 | 13 | 7 | 82 |
| **Clinical Frailty Scale** |  |  |  |  |  |  |
| 1-3 | 80 (14.3) | 286 (51) | 108 (19.3) | 2 (0.4) | 85 (15.2) | 561 (35.4) |
| 4-5 | 135 (35.8) | 144 (38.2) | 40 (10.6) | 25 (6.6) | 33 (8.8) | 377 (23.8) |
| 6-9 | 218 (34.3) | 171 (26.9) | 40 (6.3) | 192 (30.2) | 14 (2.2) | 635 (40.1) |
| Missing | 0 | 1 | 1 | 0 | 9 | 11 |

**Supplementary Table 2: Crude and Multivariable Cox proportional hazards regression presenting crude, and adjusted Hazard Ratio (HR) for the base model for length of stay**

|  | **Crude HR (95% CI)** | **p value** | **Adjusted HR^&^ (95% CI)** | **p value** |
| --- | --- | --- | --- | --- |
| **Sex** |  |  |  |  |
| Female | Reference |  | Reference |  |
| Male | 1.02 (0.90-1.17) | 0.747 | 0.94 (0.81-1.10) | 0.442 |
| **Age** |  |  |  |  |
| ≤64 | Reference |  | Reference |  |
| 65–74 | 0.75 (0.63-0.89) | 0.001 | 0.81 (0.66-0.99) | 0.044 |
| 75–84 | 0.64 (0.53-0.76) | <0.001 | 0.76 (0.61-0.96) | 0.022 |
| 85+ | 0.50 (0.41-0.62) | <0.001 | 0.60 (0.46-0.80) | <0.001 |
| **Smoking** |  |  |  |  |
| Never smoked | Reference |  | Reference |  |
| Ex-smoker | 0.95 (0.83-1.09) | 0.489 | 1.11 (0.94-1.30) | 0.223 |
| Current smoker | 1.00 (0.78-1.30) | 0.974 | 0.90 (0.67-1.22) | 0.499 |
| **Diabetes** |  |  |  |  |
| No | Reference |  | Reference |  |
| Yes | 0.88 (0.76-1.03) | 0.112 | 0.91 (0.77-1.08) | 0.277 |
| **Coronary Artery Disease** |  |  |  |  |
| No | Reference |  | Reference |  |
| Yes | 0.90 (0.76-1.07) | 0.250 | 0.97 (0.79-1.19) | 0.738 |
| **Hypertension** |  |  |  |  |
| No | Reference |  | Reference |  |
| Yes | 0.89 (0.71-1.12) | 0.337 | 1.13 (0.87-1.46) | 0.372 |
| Yes (on treatment) | 0.95 (0.82-1.09) | 0.435 | 1.10 (0.93-1.29) | 0.268 |
| **CRP** |  |  |  |  |
| <40 | Reference |  | Reference |  |
| ≥40 | 1.11 (0.97-1.28) | 0.126 | 1.13 (0.96-1.32) | 0.138 |
| **eGFR** |  |  |  |  |
| ≥60 | Reference |  | Reference |  |
| 45–59 | 0.85 (0.69-1.06) | 0.155 | 0.99 (0.78-1.26) | 0.938 |
| 30–44 | 0.83 (0.66-1.04) | 0.104 | 1.14 (0.88-1.47) | 0.317 |
| <30 | 0.88 (0.69-1.11) | 0.271 | 1.01 (0.78-1.32) | 0.915 |
| **Albumin** |  |  |  |  |
| ≥35 | Reference |  | Reference |  |
| <35 | 0.62 (0.53-0.71) | <0.001 | 0.60 (0.51-0.70) | <0.001 |
| **COPD** |  |  |  |  |
| No | Reference |  | Reference |  |
| Yes | 0.96 (0.78-1.18) | 0.697 | 1.01 (0.79-1.28) | 0.959 |
| **Heart Failure** |  |  |  |  |
| No | Reference |  | Reference |  |
| Yes | 0.78 (0.61-0.99) | 0.041 | 1.00 (0.76-1.30) | 0.987 |
| **Clinical Frailty Scale** |  |  |  |  |
| 1-3 | Reference |  | Reference |  |
| 4-5 | 0.65 (0.55-0.77) | <0.001 | 0.71 (0.58-0.87) | 0.001 |
| 6-9 | 0.50 (0.42-0.59) | <0.001 | 0.51 (0.42-0.64) | <0.001 |
| **Living arrangement** |  |  |  |  |
| Lives alone | Reference |  | Reference |  |
| Lives with 1 other | 1.31 (1.11-1.55) | 0.002 | 1.06 (0.89-1.28) | 0.507 |
| Lives with multiple others (not in a residential or nursing home) | 1.56 (1.25-1.95) | <0.001 | 1.23 (0.96-1.56) | 0.101 |
| Lives with multiple others (in a residential or nursing home) | 1.08 (0.85-1.37) | 0.519 | 1.41 (1.08-1.84) | 0.012 |

^&^Adjusted by: age; sex; smoking diabetes; coronary artery disease; hypertension; CRP; eGFR; albumin; COPD; heart failure; and frailty.

Note: the number of observations excluded due to missing data was 227
